# PROMIS® General Life Satisfaction scale: construct validity in musculoskeletal pain patients

**DOI:** 10.1186/s12998-020-00320-x

**Published:** 2020-06-16

**Authors:** Brett Vaughan, Jane Mulcahy, Kylie Fitzgerald

**Affiliations:** 1 Department of Medical Education, University of Melbourne, Melbourne, Australia; 2 College of Health & Biomedicine, Victoria University, Melbourne, Australia; 3 School of Health & Biomedicine, RMIT University, Melbourne, Australia

**Keywords:** Item response theory, Reliability estimation, Patient report outcome measure, Internal consistency, Osteopathic medicine, Osteopathy

## Abstract

**Background:**

Life satisfaction is part of subjective well-being. Measurement of life satisfaction is undertaken using self-report measures. This study aimed to evaluate the structural validity, concurrent validity, and internal structure of the PROMIS® General Life Satisfaction Scale (GLSS) in a musculoskeletal pain cohort.

**Method:**

Consecutive new patients attending the Victoria University Osteopathy Clinic (Melbourne, Australia) were invited to complete the GLSS prior to their initial consultation. Structural validity and internal structure were explored using confirmatory factor analysis and Mokken scale analysis. Concurrent validity was evaluated against a single-item measure of life satisfaction.

**Results:**

The PROMIS® GLSS comprised a single factor and formed an acceptable Mokken scale in this population. No differential item functioning was observed. A large positive correlation (*r* = 0.70) was observed between the General Life Satisfaction scale and a single-item measure of life satisfaction.

**Conclusions:**

The PROMIS® General Life Satisfaction scale demonstrated acceptable internal structure and structural validity in a musculoskeletal pain population. Additional research is required to explore concurrent validity and other measurement properties, however initial data suggests the measure could be a feasible screen of life satisfaction for Australian osteopathic patients.

## Background

Subjective well-being is a broad psychological construct with satisfaction with life (SWL) being one of the sub-constructs [1]. The latter relates to the subjective cognitions and judgments we make about our lives [2]. People form judgments of how satisfied they are based on their perception of emotional experience, with the number of positive experiences having a greater impact on higher ratings of SWL than negative emotions [3]. High levels of SWL are positively associated with a range of physical and mental health issues and health behaviours [4–8].

As SWL has previously been linked to mental and physical health status, measurement of life satisfaction may assist with patient management. Measuring life satisfaction is undertaken through self-report measures. There are a range of life satisfaction measures published in the literature with reported validity and reliability [2, 9]. The most commonly utilised of these measures is the Satisfaction with Life Scale (SWLS) [2]. From a measurement perspective, a number of SWL measures have been shown to comprise a single dimension or construct [10–12]. Measurement variance of the SWLS has also been evaluated. Emerson, Guhn and Gadermann [12] suggest that gender has little systematic influence on responses to the SWLS items however, age and culture may result in differing interpretations and subsequent responses. The most recently developed SWL measure is the PROMIS® Short Form v1.0 - General Life Satisfaction 5a scale (1Dec2017) (GLSS).

The Patient–Reported Outcome Measurement Information System (PROMIS®) (www.nihpromis.org) has been developed by the National Institute of Health (NIH) to develop, validate, and standardize an array of patient-reported outcome measures [13]. The PROMIS collection of measures encompasses physical, social and mental domains of health identified by the World Health Organisation (WHO). To our knowledge the GLSS has not been used to investigate the life satisfaction of patients seeking care for musculoskeletal complaints and to date, there is little published on its measurement properties. The purpose of this study was to evaluate the construct validity of the structure of the GLSS in this population, following the Consensus-based Standards for the Selection of Health Measurement Instruments (COSMIN) guidelines [14].

## Methods

### Participants

Patients presenting for their initial consultation at a student-led osteopathy clinic were invited to participate in the study. The clinic is located on the Victoria University campus in the Melbourne central business district, and is a clinical training environment for osteopathy students completing the final 2 years of their five-year program. All new patients were invited to complete a demographic and health information form prior to their consultation between January 1 and June 30, 2018. Consent to participate was taken as having completed the questionnaire and not indicating to ‘opt-out’ of the study. Responses from patients under the age of 18 were excluded.

### Measures

Patients completed a personal and health information questionnaire. This questionnaire was designed to collect information about a range of health behaviours and current health status consistent with data collected in Australian population health surveys [15]. Patients were also invited to complete two measures of life satisfaction. First was a single life satisfaction question *How satisfied are you with your life?* rated on an anchored Likert-type scale from 0 (not at all satisfied) to 5 representing (extremely satisfied) [16]. Single item SWL measures have been shown to be both reliable [17] and valid [18]. The second measure was the GLSS comprising five items rated on a 7-point Likert-type scale from 1 (Strongly disagree) to 7 (strongly agree).

### Data analysis

Each new patient form and questionnaire was screened and relevant data (additional demographics and clinical information) extracted from the clinical history by a single author (BV) then de-identified. Data from each form was entered into SPSS (IBM Corp, USA) [19] for analysis then exported to Microsoft Excel. The GLSS was scored using the Health Measures Scoring Service (https://www.assessmentcenter.net/ac_scoringservice) and results entered into Excel. The Scoring Service generates a T-score, the total score for the measure converted to a standardised score. A score of 50 is the mean T-score for the American general population for the GLSS [20].

The *R* program [21] was used to perform the analyses. Data missing completely at random (MCAR) were imputed using the *twoway* function in the *TestDataImputation* [22] package prior to analysis. Descriptive statistics were generated for demographic variables using the *psych* package [23]. Multiple statistical approaches were used to evaluate the measurement properties of the GLSS: confirmatory factor analysis (CFA), reliability estimations, Mokken scale analysis and differential item function. Concurrent validity of the GLSS was evaluated by way of a correlation coefficient (Pearson’s *r*) with the single-item life satisfaction question.

Confirmatory factor analysis has been performed together with Mokken scaling in numerous studies [24–28]. Authors suggest the combination of these two approaches (classical test theory and modern test theory respectively) may provide complimentary measurement property data [24, 28] (for example dimensionality [25, 29]) and provide data that allows for comparison with other research [24, 30]. Boothroyd, Dagnan and Muncer [31] also suggest that “Mokken analysis can be used in a confirmatory way to check whether a proposed scale is acceptable” (p. 533). In the case of the current work the additional benefit of utilising both data analysis strategies was to provide measurement data in a population where the questionnaire has not been utilised.

#### Confirmatory factor analysis

Confirmatory factor analysis (CFA) was performed using the *lavaan* package [32]. Given the GLSS data are ordinal, the robust weighted least squares (WLSMV) estimation method was used [33]. Multiple CFA fit statistics were generated given the varied measurement properties of each fit statistic [34, 35] and the recommended cut values are described in Table 3.

#### Reliability estimations

Internal structure of the GLSS was also evaluated using McDonald’s *omega* total and hierarchal [36, 37], in addition to the ordinal Cronbach’s *alpha* [38] statistic. Confidence intervals (95%) were calculated for the *omega* total and *alpha* statistics. McDonald’s omega hierarchal values over 0.5 provide additional support for a total score calculation [39].

#### Mokken scale analysis

Mokken scaling analysis (MSA) is a non-parametric item response theory (IRT) statistical approach with scale construction for polytomous items based on four assumptions: the scale measures a single latent construct (unidimensionality); higher levels of the latent construct correspond with higher values selected for individual items (monotonicity); responses to one item should not be systematically influenced by responses to another item (local dependence) [40]; and, non-overlapping item characteristic curves (non-intersection) [41]. A Mokken scale is constructed when these assumptions are met.

The Mokken scale analysis was performed using the *mokken* package [42] and the following steps describe the analysis:

1. Evaluate items that may form Mokken scales using the automated item search function (aisp) [43]. Initial cut-off was set at 0.3 then retested at 0.5 increments until the scales could not be explained.
2. Calculation of the scalability coefficient(s) for all items creating a scale (H), the individual items (Hi), and item pairs (Hij). Interpreted as < 0.3 = ‘weak’, 0.4–0.5 = ‘moderate’, > 0.5 = ‘strong’.
3. Local dependence was evaluated with the conditional association procedure [44]. For locally dependent items, the item with the lower Hi value was removed and the data set reanalysed.
4. Graphical and numerical approaches were used to evaluate monotonicity to ensure that each item demonstrated item response functions that monotonically increased.
5. Invariant item ordering (H^T^) was then evaluated with values < 0.3 suggesting the items could not be meaningfully ordered, 0–3-0.4 the items may be meaningfully ordered, items of between 0.4–0.5 demonstrating moderate ordering, and items with H^T^ greater than 0.5 demonstrating strong item ordering [45].

Once a scale had been finalised, Mokken’s *rho* was evaluated as a reliability estimation with a value over 0.7 being acceptable [46].

#### Differential item function

Differential item function (DIF) is used to explore whether a factor (i.e. age, gender) influences responses to an item or items on a measure in either a systematic (uniform) or non-systematic (non-uniform) manner [47]. Lack of DIF is a requirement to establish measurement invariance [47]. Age, gender, birthplace and stage of presenting complaint were the factors explored in the current work. Analysis of DIF was undertaken in the *lordif* package [48] using the likelihood ratio chi-square test and an alpha value of *p* < 0.01.

## Results

Six hundred and thirty-two (*N* = 632) patients attended the clinic during the data collection period. Two hundred and twelve (*n* = 212, 33.5%) did not provide data or declined to participate, with nine (1.8%) additional patients excluded as they were under 18 years of age. No data was collected on non-participants. Data from 411 (65%) were available for analysis. Age, gender and clinical characteristics for the sample are in Table 1. Descriptive statistics for the GLSS are presented in Table 2. The single-item life satisfaction question mean was 3.92 (±0.83) and a median of 4 [IQR 4–4].

**Table 1:** Demographic data for patients participating in the study

Gender	
Male	164 (39.9%)
Female	247 (60.1%)
Age	
Mean (±SD) years	33.47 (±13.2)
Range	18–80 years
Median	29 years
Stage	
Acute	183 (44.5%)
Chronic	227 (55.2%)
Region of presenting complaint	
Spine & pelvis	238 (57.9%)
Upper extremity	61 (14.8%)
Lower extremity	98 (23.8%)

Note: percentages that do not add to 100% represent missing data

**Table 2:** Descriptive statistics and correlation for the PROMIS General Life Satisfaction 5a scale items and total score

PROMIS item	Mean (SD)	Median [IQR]	Range	SWL correlation [95%CI]	Beta^a^	SE	*p*-value
PA045m. In most ways, my life is close to perfect	4.92 (1.47)	5 [4–6]	1–7	0.64 [0.58, 0.69]	0.81	0.02	< 0.01
PA046. If I could live my life over, I would change almost nothing	4.90 (1.63)	5 [4–6]	1–7	0.53 [0.46, 0.60]	0.93	0.01	< 0.01
PA047. I am satisfied with my life	5.68 (1.22)	6 [5–6]	1–7	0.69 [0.63, 0.74]	0.93	0.01	< 0.01
PA048. So far I have gotten the important things I want in life	5.52 (1.30)	6 [5–6]	1–7	0.58 [0.51, 0.67]	0.76	0.02	< 0.01
PA049m. My life situation is excellent	5.53 (1.18)	6 [5–6]	1–7	0.65 [0.59, 0.70]	0.85	0.01	< 0.01
Total score	26.57 (5.82)	28 [24–30]	5–35	0.70 [0.65, 0.74]			
T-score	54.32 (8.90)	55.20 [49.27–58.30]	23.0–73.4	0.70 [0.65, 0.74]			

*SWL* single item satisfaction with life score, ^a^standardised estimate, *SE* standard error

Confirmatory factor analysis fit statistics for the one factor model are presented in Table 3 and item statistics presented in Table 2. These results suggest a fit of the data to a one-factor model representing the latent construct of life satisfaction.

**Table 3:** Confirmatory factor analysis statistics PROMIS General Life Satisfaction 5a

Statistic	Recommended value	Model
χ2	NA	33.77
χ2 *p*-value	> 0.05	< 0.01
df	NA	5
χ2/df	< or = 2	6.75
Comparative fit index (CFI)	> or = 0.9	0.998
Tucker-Lewis index (TLI)	> or = 0.9	0.997
Root mean square residual (SRMR)	As close to 0 as possible	0.034
Root mean square error of approximation (RMSEA)	< or = 0.08	0.118 (CI 0.083–0.158)

**Fig. 1:**
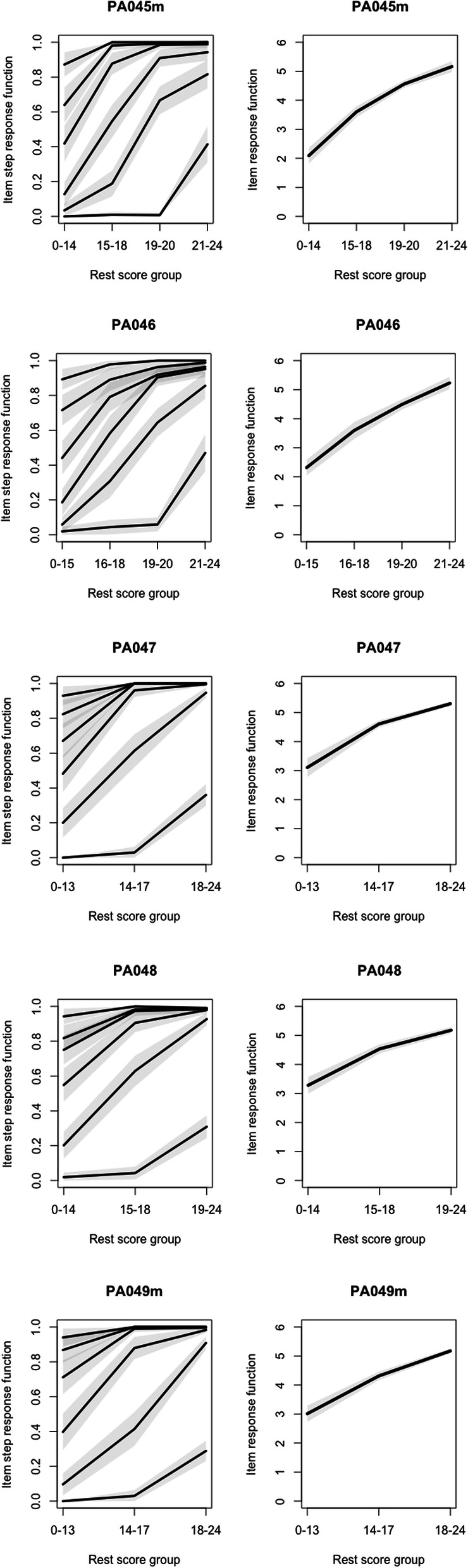
Graphical evaluation of PROMIS General Life Satisfaction 5a scale items

Results of the MSA suggest the GLSS forms a strong Mokken scale in the current population, with acceptable Hi coefficients, no monotonicity violation (Fig. 1) and *low* accuracy of item ordering (Table 4). Reliability estimations were also acceptable (Table 4). The results provide support for the GLSS being a unidimensional measure in this population. The sample size prevented further MSA to examine the internal structure of the GLSS for demographic variables such as gender. These were subsequently evaluated using a differential item function analysis. Variables were dichotomised for the DIF analysis: age (< 30 years/30 years or older), gender (male/female), stage (acute/chronic), and born in Australia (yes/no).

**Table 4:** Mokken scaling coefficients and reliability estimations for each of the PROMIS General Life Satisfaction 5a items and scale

Coefficient H	0.700 (±0.031)
Item coefficients (H*i*)	
*In most ways, my life is close to perfect (PA045m)*	0.657 (±0.042)
*If I could live my life over, I would change almost nothing (PA046)*	0.747 (±0.028)
*I am satisfied with my life (PA047)*	0.748 (±0.031)
*So far I have gotten the important things I want in life (PA048)*	0.633 (±0.038)
*My life situation is excellent (PA049m)*	0.728 (±0.028)
Invariant item ordering (H^T^)	0.37
Mokken’s *rho*	0.91
Cronbach’s *alpha* (ordinal)	0.91 [95%CI 0.90–0.93]
McDonald’s *omega* total (ordinal)	0.91 [95%CI 0.90–0.93]
McDonald’s *omega* hierarchical (ordinal)	0.91

Pearson’s *r* coefficient for the correlation between the GLSS T-score and single-item SWL measure was 0.70 (95%CI 0.65, 0.74]) suggesting a large correlation between the scores for each measure. Correlations between the individual items and the single-item SWL score were *moderate* to *high* (Table 2).

## Discussion

The current study explored the construct validity of the PROMIS® General Life Satisfaction scale in a musculoskeletal pain population and identified the measure demonstrated acceptable measurement properties in this cohort. A further aim evaluated the concurrent validity of a single item life satisfaction question and the PROMIS General Life Satisfaction scale. The mean T-score was 54.32 (+/− 8.90) suggesting the average life satisfaction across the cohort is consistent with American general population data (the comparator when using PROMIS scales) [49]. At the time of writing, there was no Australian data using this measure, therefore it is not possible to draw comparisons. The PROMIS scales are anchored with a mean of 50, therefore the current population displays a slightly higher level of life satisfaction than the American general population.

Classical test theory (CTT), in this case CFA, was used to evaluate the construct validity of the GLSS. A number of CFA fit statistics support the presence of a single factor representing the latent construct of SWL, including the CFI, TLI and SRMR. These relative fit statistics are less affected by sample size compared to the chi-square test [50]. The other fit statistics were less positive in their support of the single factor. The chi-square test was statistically significant, meaning we accept that the one factor model fits perfectly. However, statistical significance may be due to the chi-square test being suited to more complex models compared with the one-factor model in the current work [50, 51]. Kenny [51] also suggests chi-square will always be statistically significantly with sample sizes greater than 400. The RMSEA value also suggests the one-factor model may not be ‘close-fitting’ however this is likely due to the calculation of this statistic relying on the degrees of freedom (*df*) and the aforementioned chi-square. Small *df* values result in greater sampling error and a resultant high RMSEA value [51]. Ordinal Cronbach’s *alpha* and McDonald’s *omega* reliability estimations also support the single factor. These results are consistent with other SWL research suggesting that many life satisfaction measures evaluate a single latent construct [10–12]. The current study is also the first to provide confirmatory factor analysis and reliability estimation data for the GLSS and offers a useful comparator for future research.

Mokken scale analysis (MTT) was utilised to evaluate the internal structure and dimensionality of the GLSS. This non-parametric item response theory approach suggests that the GLSS is unidimensional, with acceptable scale measurement properties. The unidimensional nature of the scale is further supported by the high McDonald’s omega hierarchal value [39, 52], with the coefficient suggesting that 91% of the variance of the GLSS T-Score is due to the general factor – life satisfaction. The other MSA results support the GLSS as meeting the requirements of the Mokken scale [53]. The ability to order the GLSS items (invariant item ordering), that is, the order of the items consistently reflects an increasing level of life satisfaction, does not appear possible. This suggests that some patients may report high levels of satisfaction with an item whereas for other items they will report it as low, even if their overall life satisfaction T-score is the same.

This work also provides additional evidence for the GLSS with respect to differential item function. Age and culture have been shown to influence responses to life satisfaction measures [10–12]. The current work did not identify DIF for age, gender, being born in Australia nor the stage of the presenting complaint. Lack of DIF for age may be a reflection of the younger population in the current work, and this study provides support for the notion that gender does not systematically influence SWL item responses. That said, typically only one of the groups in the DIF analysis reached the 200 per group sample size suggested by Scott, Fayers, Aaronson, Bottomley, de Graeff, Groenvold, Gundy, Koller, Petersen and Sprangers [54] to achieve approximately 80% power. Further, we chose not to explore DIF for region of complaint as Bonferroni-adjusted *p*-values would likely require more than 500 responses in each complaint group [54] which is prohibitive in the current work.

A large positive correlation was observed between the single-item SWL question and the GLSS scores supporting the criterion validity of the measures. These two measures exhibit a shared variance of approximately 50% suggesting significant overlap in construct measurement. Similar shared variances have been described in studies exploring the Satisfaction with Life Scale and single item SWL measures [18, 55]. Although single item measures are widely used in large scale studies, Diamantopoulos, Sarstedt, Fuchs, Wilczynski and Kaiser [56] suggest that they only be used in small scale studies (less than 50 participants) and that multi-item measures will perform more appropriately where larger samples are used. This work by Diamantopoulos, Sarstedt, Fuchs, Wilczynski and Kaiser [56] was based on simulation data so further work in real populations is required. Our work highlights the need for additional work on the concurrent validity of the GLSS, particularly with other multi-item life satisfaction measures.

There are several limitations in the current work. The study used a consecutive sampling design. As such non-response, acquiescence and social desirability biases [57] may influence responses to the GLSS. Non-response was an issue as one-third of patients presenting during the study did not complete the measure. This may have been a result of all participants attending as new patients, with other data being collected on the same health demographics form. Using the GLSS in isolation for returning patients or with only a small number of other measures may have resulted in a higher response rate. The study was undertaken in a student-led clinical teaching environment and the patient cohort may not be reflective of those who present for musculoskeletal care in other manual therapy clinical environments in Australia.

## Conclusion

The present study has provided evidence to support the internal structure and dimensionality of the GLSS in a population seeking musculoskeletal care. The study also provided some evidence to support the use of a single-item SWL question, although this question may not capture the breadth of the construct. The GLSS demonstrates elements of construct and criterion validity that support its utility as a measure of life satisfaction in Australian musculoskeletal care populations. Further research is now required to evaluate other aspects of reliability, validity and responsiveness of the GLSS and also evaluate the measure in other Australian clinical populations.

## Data Availability

The datasets generated during and/or analysed during the current study are available in the *figshare* repository, 10.26188/5e97cb9a362e7.
